# Prioritizing the Risk of Multiple Invasive Species in the Semiarid Rangelands of Iran: An Ecological Approach to Multicriteria Decision‐Making

**DOI:** 10.1002/ece3.71287

**Published:** 2025-05-22

**Authors:** Hossein Bashari, Fereshteh Bazgir, Mohammad Reza Vahabi

**Affiliations:** ^1^ Department of Natural Resources Isfahan University of Technology Isfahan Iran

**Keywords:** analytic hierarchy process, biological invasion, cluster analysis, ordination, rangeland ecosystems

## Abstract

Invasive plants pose a threat to production sustainability due to their detrimental effects on soil, food cycles, and hydrology. This study aimed to identify and analyze the effects of five invasive plant species on the rangelands of western Isfahan province, Iran. A random‐systematic sampling of vegetation cover and soil was conducted at four rangeland sites, and mean soil characteristics were compared using one‐way analysis of variance and Tukey's test. Parametric principal component analysis (PCA) and nonparametric multidimensional scaling (NMDS) analysis in CANOCO and PATN software were used to investigate the relationship between environmental factors and vegetation cover. Cluster analysis was employed for habitat grouping, and the Analytic Hierarchy Process (AHP) was utilized to analyze the risk of invasive plants. The analysis involved three main criteria, eight subcriteria, and five options. The compatibility ratio of each criterion was calculated using Expert Choice software to assess the accuracy of criteria weighting. Parametric ordination revealed significant correlations between the first and second principal components and mean annual precipitation, mean annual temperature, altitude, slope, nitrogen, and calcium. NMDS analysis revealed significant correlations between plant species and seven environmental variables in a three‐dimensional ordination space (*p* < 0.05). Among the target species, *Eryngium billardieri* showed a positive correlation with rainfall, altitude, slope, calcium, nitrogen, and a negative correlation with mean annual temperature, rock, and gravel. However, the relationship of other species with environmental factors was not significant. Notably, *Cousinia bachtiarica*, *Eryngium billardieri*, *Phlomis persica*, *Euphorbia decipiense*, and *Poa bulbosa* exhibited the most destructive effects, respectively. The study results can inform targeted efforts to protect rangeland ecosystems against invasive plants. Furthermore, the study method is applicable for assessing the risk of other plant species in semiarid ecosystems.

## Graphical abstract

This study examines the ecological dynamics of five invasive plant species in western Isfahan province, Iran, investigating their relationship with environmental factors through various analytical techniques. Significant correlations between soil characteristics, elevation, mean annual precipitation, and vegetation distribution were observed, informing tailored management strategies. By prioritizing invasive species based on impacts and potential for spread, targeted control measures can be implemented to mitigate ecological and economic impacts, fostering sustainable rangeland ecosystems.

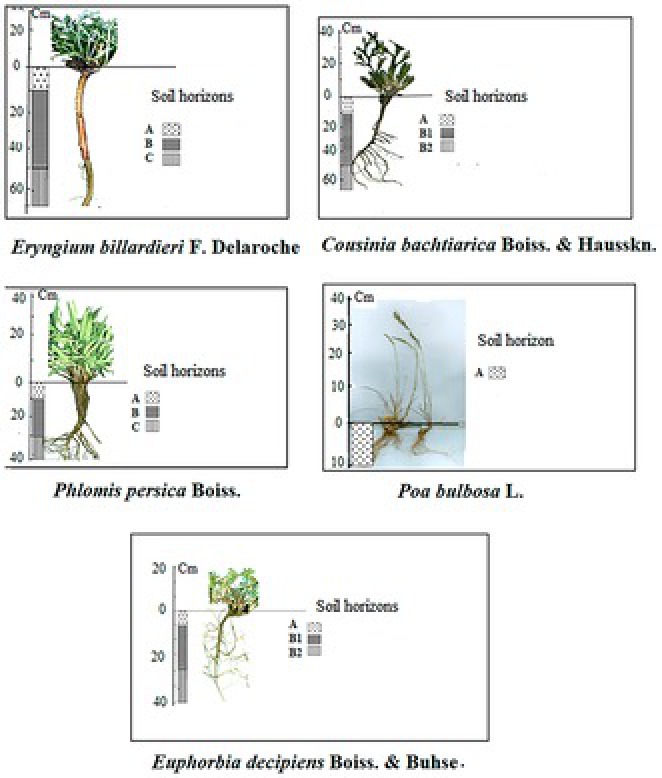

## 1 Introduction

Global environmental changes, including alterations in land use, shifts in climate patterns, and escalating carbon emissions from activities such as fossil fuel consumption and deforestation, have resulted in significant ecological transformations worldwide (Foley et al. 2005). Among the many consequences of these changes, the invasion of nonnative species into new habitats has emerged as a pressing concern (CBD 2014). Invasive species, with their detrimental effects on natural and agricultural environments, human well‐being, and biodiversity, pose a substantial threat to global ecosystem stability (Sala et al. 2000; Simberloff et al. 2012).

This threat is not confined to Iran; invasive species are also a major concern in semiarid and arid ecosystems across the globe, including in regions of North America, Australia, and parts of Africa (Dukes and Mooney 2004; Martin et al. 2015; Early et al. 2016). Invasive species dynamics in such dryland ecosystems pose challenges to global conservation and biodiversity efforts, making this study relevant to a wide range of ecological and management contexts.

However, some studies suggest that, under certain conditions, invasive species can enhance biodiversity or ecosystem functioning by filling ecological gaps left by the loss of native species (Vilà et al. 2011; Strayer 2012). Such perspectives underscore the complexity of invasive species impacts and highlight the need for tailored management approaches that account for local ecological contexts (Mack et al. 2000).

The impacts of biological invasions extend across various dimensions of ecosystems, including changes in species diversity, disruptions in community organization, shifts in water and nutrient cycles, and reduced reproductive capabilities of native plants through pollination, germination, and seed production. Consequently, invasive species play a crucial role in driving biodiversity loss and species extinction, rendering ecosystems more vulnerable to the pressures of climate change (Vilà et al. 2011; Simberloff et al. 2012). Despite this, research by Ewel and Putz (2004) suggests that in certain circumstances, invasive species can enhance ecosystem services, particularly in degraded ecosystems, by providing stability or even aiding in soil recovery. This raises questions about the blanket characterization of all invasive species as harmful and points to the importance of context‐specific management strategies.

Invasive plants, among the diverse array of invasive organisms, exert the most significant influence, profoundly affecting ecosystem biodiversity and the availability of food for grazing animals, leading to notable economic and social losses (Pyšek et al. 2020). The expansion of invasive plants in rangelands often signals underlying management shortcomings and difficulties, resulting in changes in soil seed banks, disruptions, and reductions in ecosystem services and products (Gioria et al. 2014). As a result, identifying invasive plants and assessing their harmful effects becomes crucial in enabling rangeland managers to recognize the most damaging species and develop strategies to minimize their impact. This, in turn, substantially reduces the expenses linked to control and eradication efforts, especially in resource‐limited situations (Mostert et al. 2018).

Multicriteria decision‐making (MCDM) has become a valuable tool for prioritizing the risk of invasive plant species in various ecosystems based on a range of environmental and ecological factors. The core components of MCDM include a set of management options subject to evaluation, mutually exclusive criteria guiding the assessment of these options, scores representing how well each option performs against the criteria, and weights reflecting decision‐makers' perspectives on the significance of each criterion (Cohen et al. 2024). Among the range of MCDM techniques employed by natural resource managers, the Analytic Hierarchy Process (AHP) stands out for its ability to evaluate the consistency and coherence of decision‐makers' judgments. Additionally, when resource constraints limit the use of alternative methodologies, AHP emerges as a viable option (Khedrigharibvand et al. 2018). Extensive research has utilized the AHP process to evaluate ecological potential and guide management decisions (Grošelj et al. 2023). This technique has been instrumental in designing strategies to prioritize management actions for controlling invasive plant species in various regions, including South Africa (Mostert et al. 2018), as well as in other applications (Bahrami et al. 2024).

The AHP allows decision‐makers to break down complex decisions into a hierarchy of more easily comparable elements, such as criteria, subcriteria, and options. This structure is particularly useful in ecological management, where factors such as the ecological impact, invasion potential, and management difficulty of invasive species often involve both quantitative and qualitative data. Moreover, the method's reliance on pairwise comparisons ensures that subjective judgments about ecological risks can be incorporated into an objective framework, reducing inconsistencies in the decision‐making process. By calculating weights for each criterion, AHP provides a systematic way to rank invasive species according to their relative risks, aiding in the efficient allocation of limited resources for control efforts (Saaty 1980). Given the scarcity of resources in many rangeland management scenarios, AHP emerges as an optimal approach for prioritizing invasive species for management and control (Khedrigharibvand et al. 2018).

Central to the conduct of ecological studies and the formulation of a systematic approach to invasive species assessment is the exploration of the relationship between plant species and environmental factors, a focal point of investigation in this study utilizing various multivariate analyses. Noteworthy examples of the application of ordination and clustering methodologies in clarifying the impact of environmental factors on vegetation cover include recent studies by Thammanu et al. (2021) and Mukundi et al. (2024).

Numerous studies have primarily focused on agricultural and horticultural weeds, leaving a significant gap in our understanding of invasive plants in rangelands. This gap is especially pronounced in semiarid regions like Central Iran, where the ecological impacts of invasive species on rangeland ecosystems remain understudied. This study seeks to address this gap by examining the impacts of five invasive plant species on the semiarid rangelands of Iran. The five invasive plant species selected for this study, including *Eryngium billardieri* F. Delaroche, *Cousinia bachtiarica* Boiss. & Hausskn, *Phlomis persica* Boiss., *Euphorbia decipiens* Boiss. & Bushe, and *Poa bulbosa* L., are known for their adaptability and invasiveness in semiarid rangelands. *Eryngium billardieri*, a perennial forb from the Apiaceae family, thrives in disturbed habitats, particularly overgrazed areas, and has demonstrated opportunistic behavior, increasing its prevalence after fire events (Khajeddin and Yeganeh 2008; Mirdavoodi et al. 2013). While its medicinal properties have been widely studied (Sefidkon et al. 2004), its invasive potential is concerning, especially in regions like India, where it affects agricultural productivity (Peters et al. 2014).

*Cousinia bachtiarica* and *Phlomis persica* are both hemicryptophytic forbs that dominate under increased grazing pressure and deteriorating rangeland conditions (Shakeri Broojeni et al. 2014). Their ability to reproduce through both sexual and asexual means facilitates their rapid colonization of degraded ecosystems. Similarly, *Poa bulbosa* , a geophytic perennial grass from the Poaceae family, exhibits invasive traits through its clonal reproduction and highly competitive ability in overgrazed habitats (Novak and Welfley 1997). This suite of species, with their ecological resilience and dominance in disturbed environments, presents significant challenges to rangeland management.

This study introduces a novel integration of multivariate statistical techniques with AHP to provide a more holistic assessment. While MCDM techniques have been employed in ecological studies, their application to the prioritization of invasive species management in resource‐limited rangelands remains scarce. By combining parametric principal component analysis (PCA) and nonparametric multidimensional scaling (NMDS) ordination methods with AHP, this study delivers a comprehensive, data‐driven framework for evaluating both the ecological impacts and the prioritization of management strategies for invasive species.

The innovation of this study lies in synthesizing multiple methodologies. Previous research has often focused on individual aspects, such as spatial modeling of invasive species (Martin et al. 2015; Elith 2017), prioritization using AHP (Nielsen and Fei 2015), the autecology of invasive species (Master et al. 2020), or their relationships with environmental factors (Oh et al. 2021). However, these studies typically address isolated elements of invasive species impacts. In contrast, comprehensive approaches that combine these methods into a cohesive framework are rare, particularly in understudied regions such as central Iran and western Asia. Additionally, detailed analyses of invasive species' root systems in these regions are lacking. This multifaceted methodology, encompassing vegetation composition analysis, soil characterization, root system profiling, environmental response assessment, and species ranking, represents a significant advancement in the study of invasive species. It offers a robust framework that can be adapted for use in similar ecosystems worldwide, addressing both the ecological and management challenges posed by invasive species.

Our hypothesis suggests that these invasive plants will negatively affect soil quality, thereby endangering the sustainability of the rangelands. By employing various multivariate techniques, we aim to provide valuable insights to inform targeted conservation efforts aimed at protecting rangeland ecosystems from the harmful effects of invasive plants. Specifically, we hypothesize that the target species possess morphological traits conducive to dominance in rangeland ecosystems. We also anticipate that climate variables play a crucial role in the presence and spread of invasive plants, outweighing soil variables. Finally, we posit that prioritizing the risk posed by invasive plants is essential for maintaining rangeland integrity and implementing effective control measures against invasive species.

This study aims to investigate the ecological impacts of five invasive plant species on semiarid rangelands in Iran. Given the significant gap in research concerning invasive plants in these ecosystems, our objectives include assessing their effects on soil quality, vegetation composition, and overall ecosystem health. By employing a novel integration of multivariate statistical techniques and AHP, we aim to provide a transferable framework for evaluating invasive species in resource‐limited rangelands across the globe. By combining ecological impacts with management prioritization, our framework can be adapted to similar ecosystems facing invasive species pressures, such as those in North America and the Mediterranean. Ultimately, our research seeks to inform targeted conservation efforts, ensuring the sustainability and resilience of rangeland ecosystems.

## 2 Materials and Methods

**FIGURE 1:**
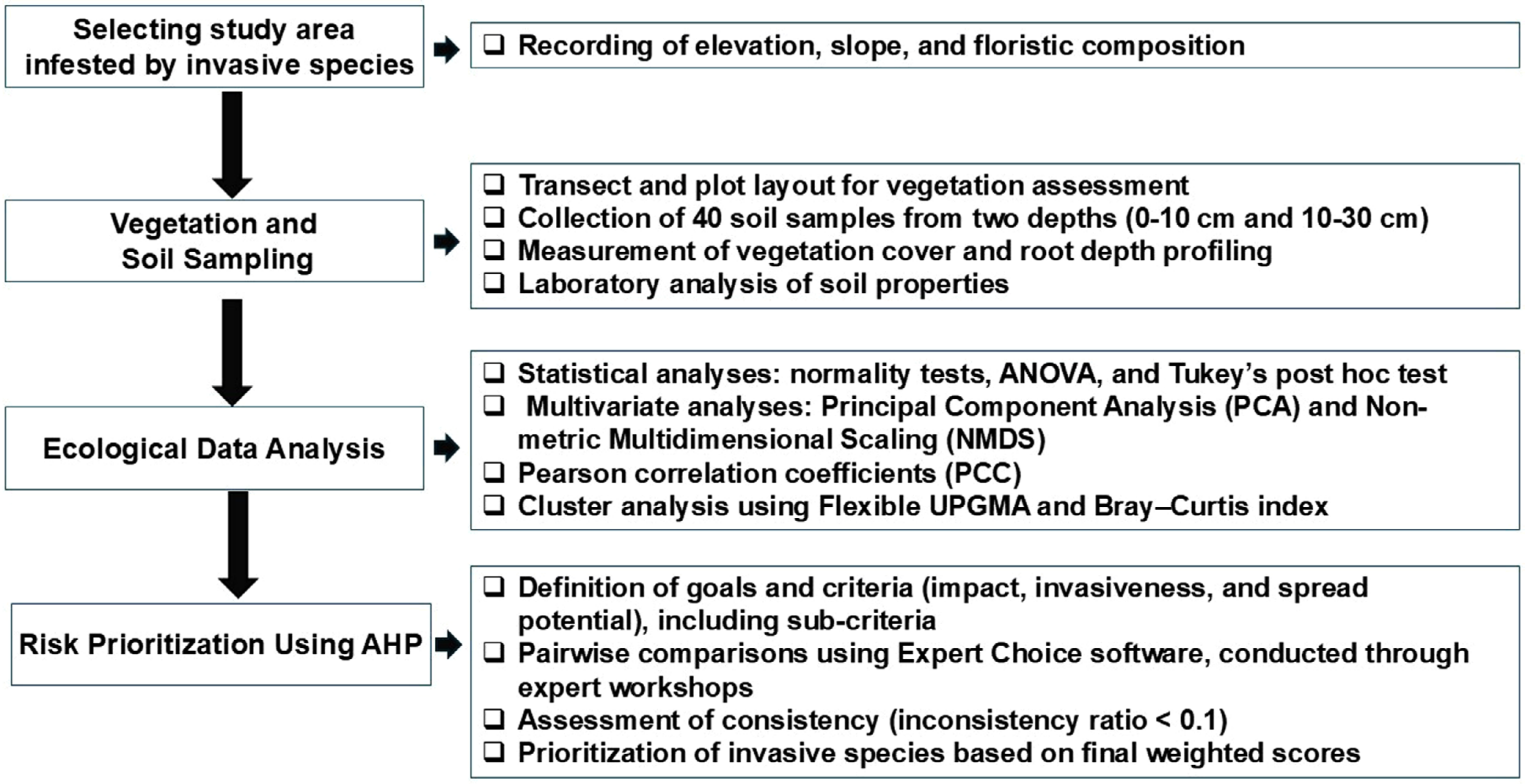
Flowchart of study: Steps in ecological analysis and risk prioritization of invasive species.

The prioritization of invasive species risk involved selecting four infested sites, conducting vegetation and soil sampling, and analyzing the data to examine the relationship between environmental factors and vegetation cover. The ecological conditions of the sites were compared, and the AHP was applied to assess and rank the risks posed by the invasive species (Figure 1). Detailed steps are outlined in the subsequent sections.

### 2.1 Study Area

**FIGURE 2:**
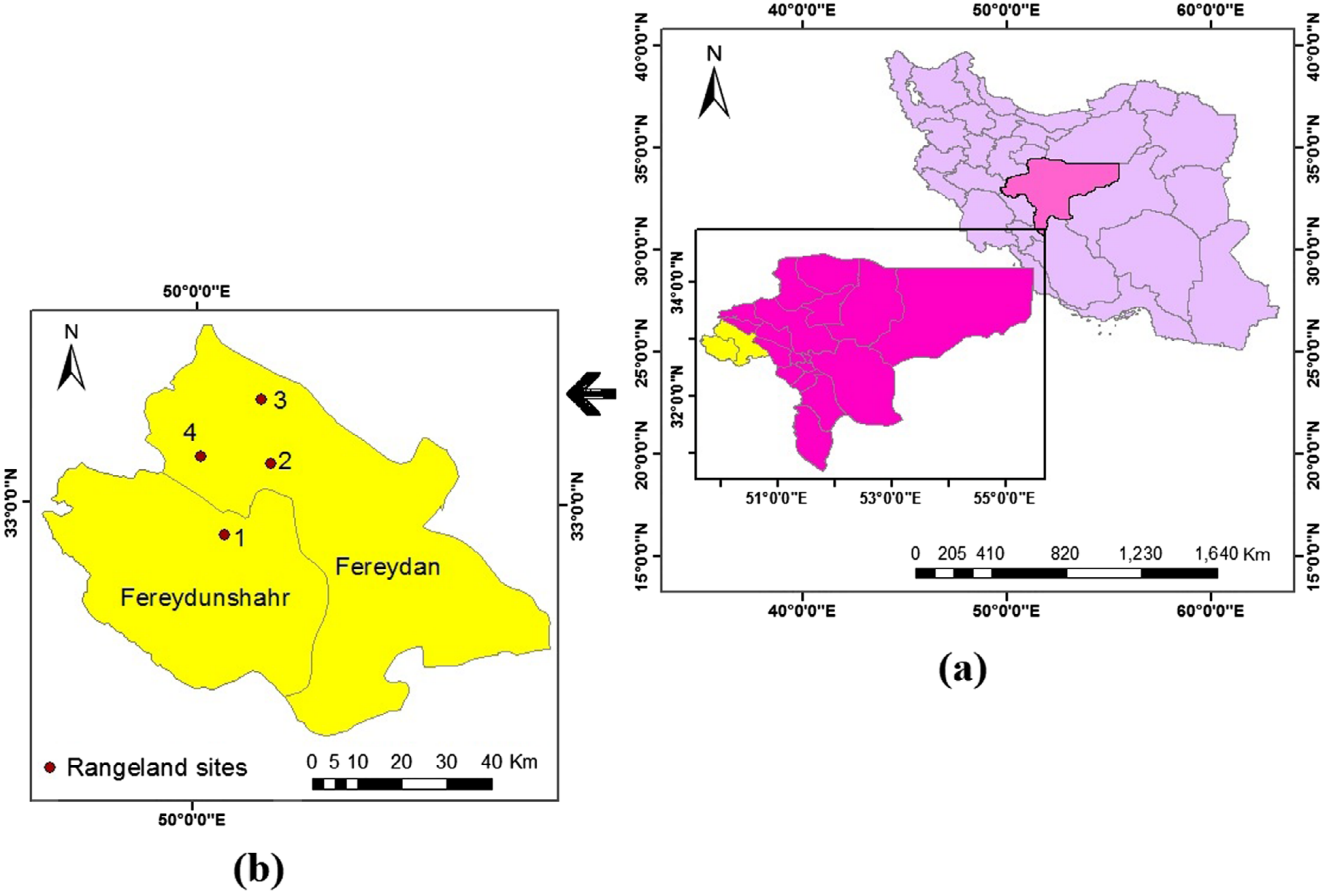
(a) Map of Isfahan Province in Iran and (b) location of sites in the study areas (site 1: Fereydunshahr, site 2: Hajfathali, site 3: Aghcheh, site 4: Noghan Olya).

This study was conducted in the central region of Iran, specifically in the semiarid rangelands of Fereydan and Fereydunshahr counties in Isfahan province (Figure 2). The average elevation of the study area is 2828 m, with a mean annual temperature of 10°Celsius and a mean annual precipitation of 542 mm. Based on Köppen and Thornthwaite climate classifications, the western region of Isfahan is categorized into semiarid cold and semiarid climate zones. The natural landscape of this region, part of the vast central Zagros Mountains, traditionally provides forage for livestock, water, and other ecosystem services to the local people. This area has a long history of grazing by sheep and goats. Overgrazing and varying severity of droughts have occurred in this region, impacting the rangeland species. Soil depth in most parts of the study area is less than 10 cm, particularly in the steep areas, with clay loam being the dominant soil texture. Notably, among the most important species present in the rangelands of the study area are *Astragalus adscendens* Boiss. & Hausskn., *Astragalus verus* Olivier, *Ferula ovina* Boiss., *Prangos ferulacea* (L.) Lindl., and *Bromus tomentellus* Boiss.

### 2.2 Field Sampling and Analysis

Initially, four sites infested with five invasive plant species were deliberately chosen (Table 1), and data on the percentage of slope and elevation above sea level were recorded for each rangeland site. A comprehensive survey was then conducted to identify the floristic composition, vegetation cover, and distribution of invasive species (Table 2). Subsequently, systematic random sampling was employed to assess both vegetation cover and soil characteristics across the study rangeland sites (Table 2). At each site, three 100‐m transects were established, spaced 50 m apart perpendicular to the prevailing slope direction. Along each transect, five plots measuring 2 square meters each were established at 20‐m intervals to record the canopy cover of plant species. For soil sampling, 5 plots were randomly selected from a total of 15 plots where vegetation cover samples were collected in each area. Soil samples were then collected from two depths in each plot: 0–10 cm and 10–30 cm, to measure the physical and chemical properties of the soil in the region. In total, 40 soil samples were collected from 4 areas and transferred to the laboratory for analysis. Soil properties such as pH, electrical conductivity, sodium ion concentration, calcium, magnesium, available phosphorus, and available potassium were analyzed using standard methods (Carter and Gregorich 2008). Total nitrogen content was determined using the Kjeldahl method, lime percentage by the back titration method, and organic matter content through the Walkley and Black method. Microbial respiration rate (MR) was determined utilizing the closed bottle method, as outlined by Sardinha et al. (2003), employing NaOH absorption, and subsequent titration with HCl, following the procedure described by Froment (1972). Additionally, the soil texture (percentage of sand, silt, and clay) was determined using the hydrometer method in the soil laboratory (Weaver et al. 1994).

**TABLE 1:** The environmental characteristics of rangeland sites.

Site number	Site name	Longitude (°)	Latitude (°)	Elevation (m)	Slope (%)	Mean annual precipitation (m)	Mean annual temperature (°C)
1	Fereydunshahr	50°00′36″	32°53′26″	2707	20	510	13.47
2	Hajfathali	50°00′10″	33°04′35″	2690	10	450	13.60
3	Aghcheh	50°06′10″	33°02′35″	2492	14	450	13.80
4	Noghan Olya	50°03′43″	33°19′55″	2350	8	400	14.33

**TABLE 2:** List of the studied invasive plant species.

Species name	Family	Growth period	Height (cm)	Geographic distribution	Biological form	Vegetative from	Reproductive method
*Eryngium billardieri* F. Delaroche	Apiaceae	Perennial	40–80	IT	He	Forb	S., AS
*Cousinia bachtiarica* Boiss. & Hausskn.	Asteraceae	Perennial	39 <	IT	He	Forb	S., AS
*Phlomis persicsa* Boiss.	Lamiaceae	Perennial	25–50	IT	He	Forb	S., AS
*Euphorbia decipiens* Boiss. & Buhse.	Euphorbiaceae	Perennial	30 >	IT	He	Forb	S.
*Poa bulbosa* L.	Poaceae	Biennial	10–50	IT, M, ES	Ge	Grass	S., AS

Abbreviations: AS, Asexual reproduction; ES, Euro‐Siberian; Ge, Geophyte; He, Hemicryptophyte; IT, Irano‐Turanian; M, Mediterranean; S, Sexual reproduction.

Through laboratory measurements of plant dry weight, we established the ratio of root dry weight to the aerial parts of the plants under examination. Additionally, comprehensive field studies and profiling of root development depth were conducted to ascertain the extent to which roots penetrate the soil.

### 2.3 Data Analysis

Following the assessment of the normality of the physical and chemical soil characteristics data using the Anderson‐Darling test and verifying the homogeneity of variances through Levene's test, one‐way analysis of variance (ANOVA) and Tukey's post hoc test were conducted using SPSS 22 software. To determine the most suitable ordination method for illustrating the relationship between plant parameters and environmental factors, Detrended Correspondence Analysis (DCA) was initially applied. Based on the resulting gradient length of 1.8, a linear method was deemed appropriate (ter Braak and Šmilauer 2002). Consequently, PCA was selected as the preferred linear ordinationtechnique using CANOCO 4.5 software (ter Braak and Šmilauer 2002). Additionally, NMDS was employed using PATN software (Belbin 2003). To assess the similarity among plant species and sampling units, the Bray–Curtis dissimilarity index was calculated (Bray and Curtis 1957). Principal axis correlation was then applied in PATN to examine relationships between plant species and environmental variables on the ordination axes. The significance of these correlations was determined through random permutation tests with 100 permutations using the Monte Carlo method implemented within the ordination procedure in PATN (Belbin 2003). Plant species and environmental factors that showed significant correlations with the ordination axes were plotted in the ordination space. Box and whisker plots were also generated using PATN software to facilitate the interpretation of changes in plant species within clusters (Belbin 2003). Kruskal–Wallis tests (Kruskal and Wallis 1952) were performed to visualize the distribution of variables within clusters. The values obtained from the Kruskal–Wallis tests provided insights into the contribution of each factor to group separation. Cluster analysis was used to classify habitats based on the arithmetic mean of vegetation cover data. Similarity among sites was quantified using the Bray–Curtis dissimilarity index. For this purpose, the Flexible UPGMA technique, a hierarchical agglomerative clustering method, was employed. Results were presented as dendrograms. The Weighted Pair Group Method with Arithmetic Mean (UPGMA) was chosen among available hierarchical clustring options for its effectiveness in representing site relationships.

### 2.4 Prioritization of the Risk Posed by the Invasive Plants

**FIGURE 3:**
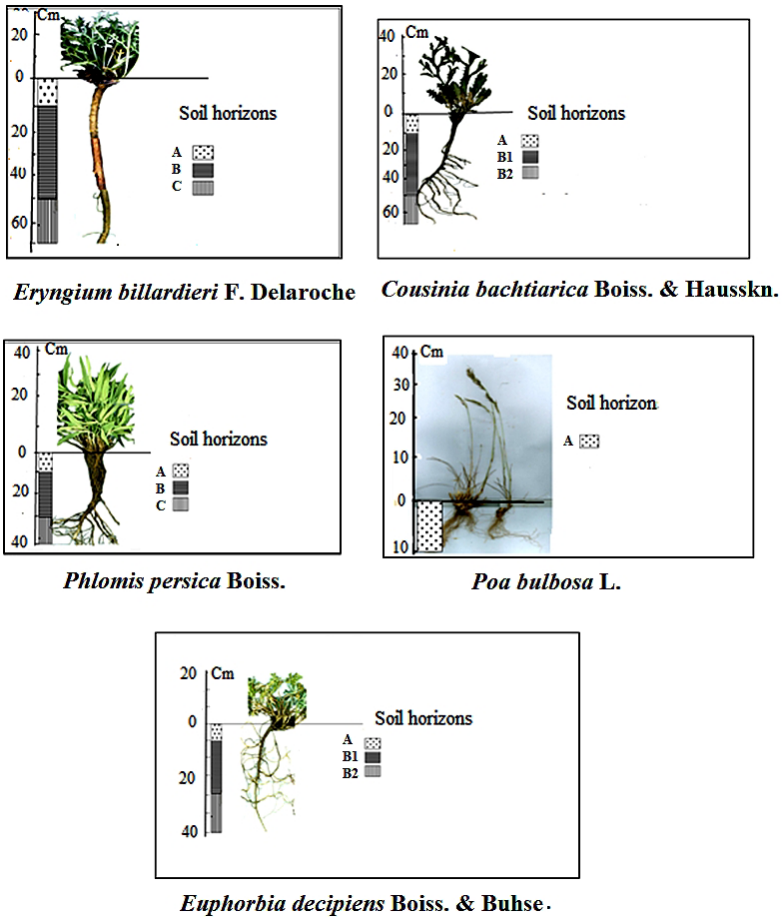
Above‐ground and below‐ground images of the invasive species under study, complemented by soil horizon details.

In our assessment of invasive plant risks, we applied AHP, a sophisticated multicriteria decision‐making method that facilitates prioritization by comparing multiple criteria in a pairwise manner to establish a preference scale among different options (Saaty 1980). This comprehensive process involves defining a goal, criteria, and subcriteria, which were carefully determined with the input of a panel comprising four experts. Additionally, we supplemented our analysis with insights from field ecological surveys and expert opinions, particularly for certain pairwise comparisons (Figure 3).

The AHP method proceeds through a series of well‐defined steps that begin with the establishment of a hierarchy, in this case, consisting of a goal (prioritization of invasive species), criteria (impact, invasion, and spread potential), subcriteria, and ultimately, the species themselves as the options. Each element in the hierarchy is compared pairwise, which requires the decision‐maker to evaluate how much more one element contributes to the goal relative to another. This method allows for the incorporation of both quantitative data (e.g., infestation percentages) and qualitative insights (e.g., expert opinions on adaptability). The pairwise comparison matrix generated from this process is then used to compute priority weights for each criterion and option, allowing for a clear ranking of invasive species risks.

In the AHP method, our focus was on assessing the criteria of impact, invasion, and spread potential of invasive species. The impact criterion evaluates the effects of invasive plants on rangeland ecosystems. Indicators representing these effects include interference or blockage of the entry of desired species, ecosystem damage, and resistance to control. The invasion criterion identifies factors influencing the invasiveness of plants, such as adaptability, competitive strength, reproduction, and dispersion. The spread potential criterion assesses the spread potential of invasive plants, considering indicators such as the percentage of rangelands infested by these species and their presence in multiple countries.

Pairwise comparison questionnaires for criteria, subcriteria, and options were input into the Expert Choice software to conduct comparisons. Criteria were evaluated pairwise based on the study's objectives, and scores were assigned to reflect their relative importance. Scores ranging from 1 to 9 were hierarchically assigned to subcriteria relative to criteria and to options relative to subcriteria.

To ensure accuracy, Expert Choice software also checks for consistency within the pairwise comparisons. Consistency in AHP is vital because it confirms that the judgments made during pairwise comparisons are logically sound. An inconsistency rate higher than 0.1 would indicate that judgment might need revision, as it would suggest contradictions in the pairwise comparisons. This iterative refinement process helps ensure that the final ranking of invasive species is both robust and scientifically grounded (Saaty 1980). Following software analysis, options were prioritized based on the assigned scores.

## 3 Results

### 3.1 Canopy Cover and Vegetation Composition of Invasive Plants

Table 3 presents the canopy cover percentage and vegetation composition of invasive plant species across four sites in central Iran. The data reveal varying levels of canopy coverage across the sites, with Fereydunshahr exhibiting the highest percentage at 28.61% and Noghan Olya the lowest at 20.08%. In terms of vegetation composition, *Eryngium billardieri* is notably prevalent across all sites, with the highest cover (15.07%) and composition percentages (52.67%) observed in Fereydunshahr. *Cousinia bachtiarica* and *Phlomis persica* also exhibit a significant presence, albeit with variations between sites. Interestingly, while some invasive species like *Euphorbia decipiens* and *Poa bulbosa* show limited coverage, they still contribute to the overall composition of vegetation.

**TABLE 3:** Canopy cover percentage and vegetation composition of invasive plant species in central Iran.

Site number	Site name	Canopy cover (%)	*Eryngium billardieri*		*Cousinia bachtiarica*		*Phlomis persica*		*Euphorbia decipiens*		*Poa bulbosa*	
			Cover (%)	Composition (%)	Cover (%)	Composition (%)	Cover (%)	Composition (%)	Cover (%)	Composition (%)	Cover (%)	Composition (%)
1	Fereydunshahr	28.61	15.07	52.67	0.00	0.00	0.00	0.00	0.60	2.10	1.90	6.64
2	Hajfathali	23.79	1.73	7.27	0.60	2.52	0.10	0.42	0.77	3.24	0.03	0.13
3	Aghcheh	27.28	0.30	1.10	2.37	8.69	0.00	0.00	1.17	4.29	0.00	0.00
4	Noghan Olya	20.08	0.60	2.99	0.20	1.00	1.83	9.11	0.00	0.00	0.37	1.84

### 3.2 Soil Parameter Disparities Among Invasive Species' Habitats

The comparison of average soil parameters across the four rangeland locations revealed consistent levels of pH, potassium (K), magnesium (Mg), phosphorus (P), and sodium (Na) at both depths (0–10 cm and 10–30 cm), as no significant differences were observed among sites. However, significant variations were observed in silt, sand, and calcium carbonate (CaCO_3_) percentages, particularly in the subsurface layer (10–30 cm). These differences were most pronounced in Noghan Olya, which exhibited distinct soil characteristics compared to Fereydunshahr, Hajfathali, and Aghcheh.

In terms of soil texture, Noghan Olya had the highest sand content (49.59%) and the lowest clay and silt percentages in the surface layer (0–10 cm), a trend that persisted in the subsurface layer (10–30 cm). Additionally, Noghan Olya exhibited the highest sodium concentration (77.32 meq/L in 0–10 cm), though this difference was not statistically significant (*p* = 0.190). At 10–30 cm, sodium levels remained variable across sites, but differences were not statistically significant (*p* = 0.492).

Furthermore, Noghan Olya had the lowest electrical conductivity (EC) in surface soil (3.73 dS/m, *p* = 0.040), further distinguishing it from the other locations. The site also exhibited notable differences in CaCO_3_ levels, with significantly lower values compared to the other rangelands (*p* = 0.020 for 0–10 cm, *p* = 0.027 for 10–30 cm). These findings emphasize the unique soil texture and chemical composition of Noghan Olya, particularly in terms of its sandy clay loam to sandy loam texture and the distribution of key variables such as CaCO_3_, sodium, and electrical conductivity (Table 4).

**TABLE 4:** Comparison of physical and chemical properties of surface (0–5 cm) and subsurface (5–25 cm) soil samples in the studied rangeland locations.

Soil variable	Unit	Soil depth	Fereydunshahr	Hajfathali	Aghcheh	Noghan Olya	*p*
Clay	%	0–10	20.93 ± 3.92^a^	31.87 ± 8.33^a^	36.52 ± 0.82^a^	27.19 ± 14.58^a^	0.056
		10–30	24.72 ± 9.10^ab^	33.56 ± 8.53^ab^	45.76 ± 3.05^a^	17.91 ± 11.28^b^	0.001
Silt	%	0–10	53.82 ± 12.80^a^	45.61 ± 3.90^a^	54.38 ± 2.71^a^	23.21 ± 9.80^b^	0.000
		10–30	42.79 ± 10.54^a^	44.54 ± 5.72^a^	47.35 ± 4.03^a^	24.58 ± 3.00^b^	0.000
Sand	%	0–10	25.23 ± 14.62^b^	22.50 ± 2.71^b^	9.09 ± 2.39^b^	49.59 ± 14.58^a^	0.000
		10–30	32.48 ± 12.30^b^	21.89 ± 2.00b^c^	6.88 ± 2.30^c^	57.51 ± 10.00^a^	0.000
Texture	—	0–10	Silt loam	Clay loam	Silty clay loam	Sandy clay loam	
		10–30	Loam	Clay loam	Silty clay	Sandy loam	
pH	—	0–10	7.58 ± 0.15^a^	7.64 ± 0.15^a^	7.70 ± 0.17^a^	7.59 ± 0.16^a^	0.633
		10–30	7.56 ± 0.15^a^	7.76 ± 0.21^a^	7.74 ± 0.08^a^	7.66 ± 0.17^a^	0.246
EC	(dS/m)	0–10	6.74 ± 2.57^a^	5.68 ± 0.52^a^	4.88 ± 1.82^a^	3.73 ± 1.03^b^	0.040
		10–30	4.43 ± 1.36^a^	4.95 ± 1.56^a^	3.53 ± 0.78^a^	3.72 ± 0.10^a^	0.198
Ca	(meq/L)	0–10	10.16 ± 2.50^a^	6.52 ± 0.52^b^	4.64 ± 1.80^b^	4.20 ± 1.03^b^	0.000
		10–30	6.18 ± 1.41^a^	6.24 ± 0.61^a^	5.28 ± 2.25^a^	4.50 ± 2.32^a^	0.391
Mg	(meq/L)	0–10	4.24 ± 1.40^a^	3.16 ± 1.73^a^	2.56 ± 0.82^a^	2.36 ± 0.80^a^	0.360
		10–30	3.68 ± 3.34^a^	3.50 ± 0.60^a^	3.00 ± 2.50^a^	2.96 ± 2.72^a^	0.120
K	(meq/L)	0–10	16.09 ± 7.75^a^	17.26 ± 8.53^a^	17.01 ± 5.26^a^	17.89 ± 11.85^a^	0.990
		10–30	13.07 ± 9.04^a^	14.54 ± 9.07^a^	10.89 ± 3.77^a^	21.27 ± 8.32^a^	0.140
P	(mg/kg)	0–10	2.90 ± 1.80^a^	2.59 ± 1.52^a^	2.86 ± 0.68^a^	2.92 ± 1.00^a^	0.970
		10–30	2.25 ± 1.50^a^	1.58 ± 0.85^a^	1.95 ± 1.09^a^	3.05 ± 0.80^a^	0.220
CaCO_3_	%	0–10	1.84 ± 0.99^a^	2.16 ± 0.57^a^	2.08 ± 0.22^a^	1.04 ± 0.21^b^	0.020
		10–30	1.42 ± 7.50^a^	2.17 ± 6.66^a^	2.09 ± 8.35^a^	0.87 ± 4.44^b^	0.027
Na	(meq/L)	0–10	26.13 ± 1.08^a^	29.96 ± 0.45^a^	52.79 ± 1.04^a^	77.32 ± 0.90^a^	0.190
		10–30	25.72 ± 0.01^a^	28.87 ± 0.04^a^	33.76 ± 0.01^a^	30.02 ± 0.03^a^	0.492
N	(meq/L)	0–10	0.24 ± 0.01^a^	0.19 ± 0.06^ab^	0.13 ± 0.01^b^	0.14 ± 0.02^b^	0.001
		10–30	0.19 ± 0.01^a^	0.20 ± 0.04^a^	0.19 ± 0.01^ab^	0.15 ± 0.03^b^	0.017
OM	%	0–10	2.82 ± 0.99^a^	2.24 ± 0.57^ab^	2.93 ± 0.22^ab^	2.05 ± 0.21^b^	0.025
		10–30	3.06 ± 0.38^a^	2.28 ± 1.02^ab^	2.36 ± 0.50^ab^	1.73 ± 0.18^b^	0.027

*Note:* Mean and standard deviation (SD, shown in parentheses) are presented in the table. Different superscript letters within each row denote significant differences between categories (Tukey's test at *p* = 0.05).

### 3.3 Root System Profiles of Invasive Species

The study investigated the root systems of the five invasive plant species, uncovering distinct traits for each. *Eryngium billardieri* showcased a robust taproot extending over 60 cm in height, with extensive development in horizons A, B, and C, a root dry weight five times that of its aerial parts (Figure 3). *Cousinia bachtiarica* presented a fibrous root system, featuring well‐developed primary and secondary roots primarily in horizons A, B1, and B2, resulting in a root‐to‐shoot ratio of 1.33. *Phlomis persica* showed a strong root system, with thin secondary roots emerging near its base and extending through soil horizons A, B, and C. The species exhibited a notable root‐to‐shoot weight ratio of 2, indicating a strong below‐ground. *Euphorbia decipiens* exhibited a shallow yet developed root system, predominantly occupying horizons A, B1, and B2, with a root‐to‐shoot weight ratio of 0.83, though comparatively weaker than *Phlomis persica*. Finally, *Poa bulbosa* had a shallow and weak root system, mainly limited to horizon A, with a root‐to‐shoot weight ratio of 0.67 (Figure 3).

### 3.4 Invasive Species Response to Environmental Factors

The results of the PCA for soil and vegetation characteristics across the four rangeland sites, dominated by invasive species, reveal that the first, second, and third principal components explain 45.4%, 35.8%, and 18.7% of the variability in vegetation cover, respectively. Upon examining the correlation between environmental factors and these principal components, the first component demonstrates a robust association with soil characteristics such as calcium and magnesium content at both soil surface and subsurface levels, electrical conductivity, nitrogen, sodium, and potassium at the soil surface, as well as average slope, elevation, precipitation, and mean annual temperature. The second component correlates with lime, clay, and sand percentages at both soil depths, along with pH at the soil surface, while the third component primarily correlates with phosphorus at the soil surface. Given that the first and second axes capture the largest share of the variation, the most influential factors for distinguishing habitats in the study areas, ranked in descending order, are soil chemical properties, physiographic characteristics, climatic properties, soil texture, and lime content.

**FIGURE 4:**
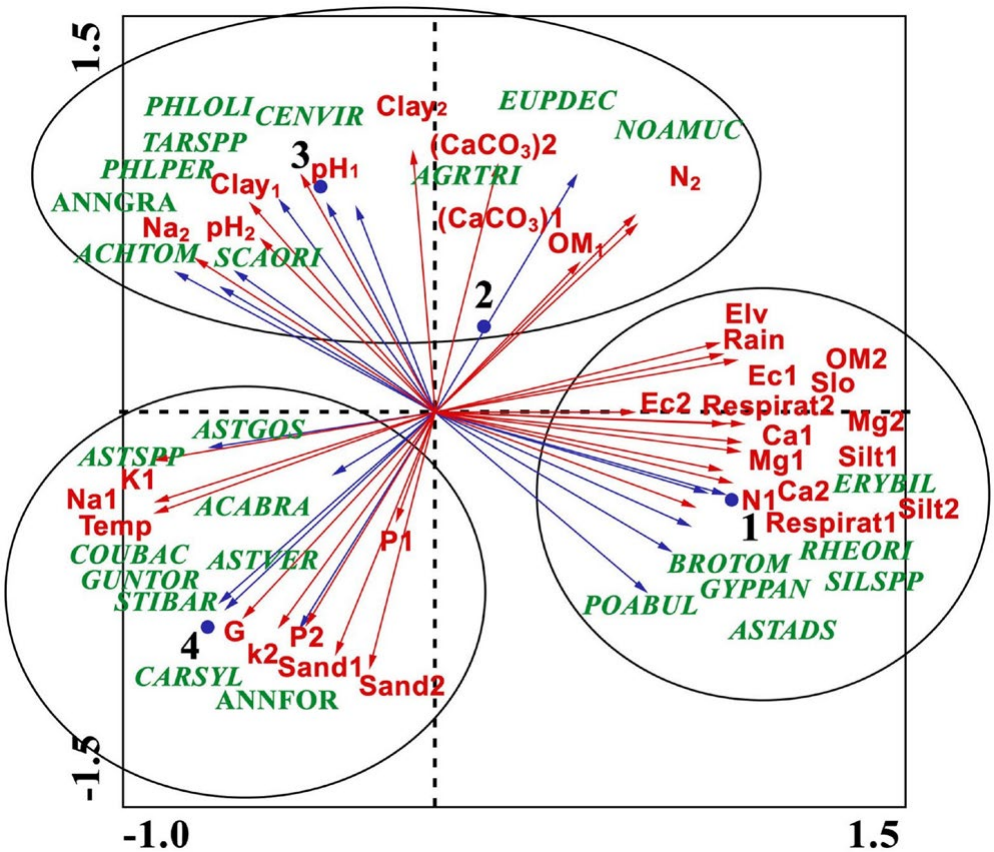
Principal component analysis‐ordination diagram of species, environment, and sampling units (species represented by green vectors, environmental factors by red vectors, and sampling units by solid circles). Sampling units 1 to 4 correspond to the rangelands of Fereydunshahr (1), Hajfathali (2), Aghcheh (3), and Noghan Olya (4). ACHTOM, *Achillea tomentosa* ; AGRTRI, *Agropyron trichophorum* ; ANNFOR, Annual forbs; ANNGRA, Annual grasses; ASTGOS, *Astragalus gossypinus*; ASTSPP, *Astragalus* spp.; ASTVER, *Astragalus verus* ; BROTOM, *Bromus tomentellus*; CARSYL, *Carex sylvatica* ; CENVIR, *Centaurea virgata* ; COUBAC, *Cousinia bachtiarica*; Elv, Elevation above sea level (meters); ERYBIL, *Eryngium billardieri*; EUPDEC, *Euphorbia decipiens*; GYPPAN, *Gypsophila paniculata* ; NOAMUC, *Noaea mucronata*; PHLOLI, *Phlomis olivieri*; PHLPER, *Phlomis persica*; POABUL, *Poa bulbosa* ; Rain, Mean annual precipitation (millimeters); RHEORI, *Rheum oriental*; SCAORI, *Scariola orientalis*; SILSPP, *Silene* spp.; Slo, Slope (percentage); temp, Mean annual temperature (degrees Celsius).

The PCA analysis reveals the presence of three distinct habitats within the study sites (Figure 4). The first distinguished habitat, encompassing site 1 (Fereydunshahr), includes two prominent invasive species: *Poa bulbosa* and *Eryngium billardieri*. Other associated species include *Bromus tomentellus*, *Noaea mucronata*, *Astragallus adscendens*, *Rheum oriental*, *Silene araratica*, and *Gypsophila paniculata*. This habitat is notable for its higher elevation, greater mean annual precipitation, and steeper slope compared to the other two habitats. Additionally, it exhibits elevated levels of organic matter, calcium, magnesium, and electrical conductivity in both surface and subsoil horizons, signifying favorable conditions for the growth and development of the identified species.

The second habitat, covering sites 2 (Hajfathali) and 3 (Aghcheh), is characterized by the predominance of the invasive species *Euphorbia decipiens* and *Phlomis persica*. Additional abundant species in this habitat include *Achillea tomentosa*, *Astragalus gossypinus*, *Acanthophyllum bracteatum*, *Scariola orientalis*, *Agropyron trichophorum*, *Centaurea virgata*, *Phlomis olivieri*, annual grasses, and *Taraxacum* sp. This habitat is defined by higher soil pH in both surface and subsoil layers and greater clay and lime content, marking it as distinct from the other two.

The third habitat, corresponding to site 4 (Noghan Olya), is characterized by a high frequency of *Cousinia bachtiarica*, although this species is also present and frequent in site 3 (part of the second habitat). This habitat hosts a diverse array of plant species, including *Astragalus verus*, *Stipa barbata*, *Carex sylvatica*, *Gundelia tournefortii*, *Astragalus* spp., and annual forbs. The surface soil contains higher levels of gravel, and this habitat experiences elevated mean annual temperatures. Additionally, both surface and subsoil horizons show increased concentrations of phosphorus, potassium, and sodium. According to the length and direction of the environmental vectors in the PCA diagram, the most influential factors shaping species distribution in this habitat are mean annual temperature, sodium content, gravel, and sand percentage.

**FIGURE 5:**
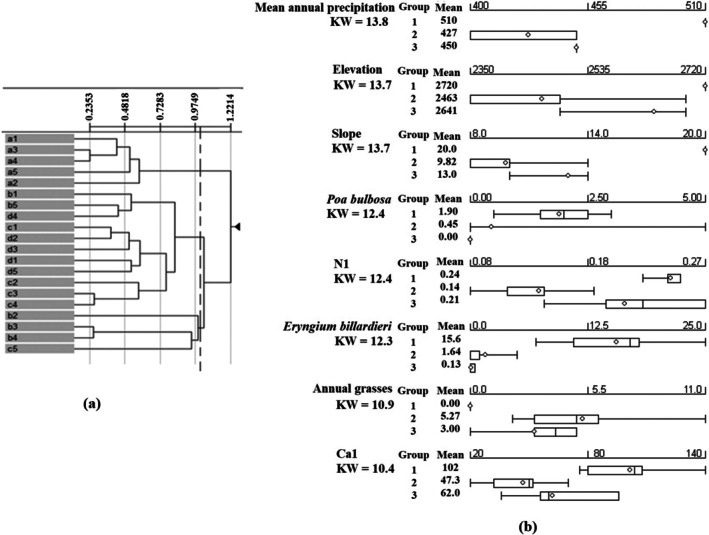
(a) Dendrogram generated from UPGMA clustering analysis of vegetation cover plots. The letters a, b, c, and d correspond to the plot symbols of sites 1, 2, 3, and 4, respectively. (b) Kruskal–Wallis statistics for environmental variables and vegetation cover percentages of plant species demonstrating significant differences across the groups.

Classification of the sites based on vegetation data also resulted in three distinct groups (Figure 5a). *Eryngium billardieri* and *Poa bulbosa* habitat located in Fereydunshahr site well match with group one. *Euphorbia decipiens* and *Phlomis persica* habitats located in Hajfathali and Aghcheh are well matched with group two. *Cousinia bachtiarica* habitat located in Noghan Olya spreads across group three.

According to the Kruskal–Wallis test, significant differences were observed among these groups in two soil variables, namely N and Ca content in the soil surface, mean annual precipitation, elevation, and slope. Moreover, the coverage percentages of *Eryngium billardieri*, *Poa bulbosa* , and annual grass varied significantly across the groups. The first group habitat, comprising habitats of *Poa bulbosa* and *Eryngium billardieri* species, exhibited higher elevation above sea level, mean annual precipitation, slope percentage, and soil calcium content compared to other habitats. The mean annual precipitation in the second group was 427 mm; however, notable variations in precipitation were observed among sites within this group. Additionally, these sites had a higher percentage of annual grasses compared to other groups. The average elevation of sites above sea level in the third group was 2641 m, and the presence of *Poa bulbosa* species was absent in this group of sites. The findings from the classification analysis and Kruskal–Wallis test corroborated the results of ANOVA and Tukey's post hoc mean comparisons (Figure 5b, and Table 4).

**FIGURE 6:**
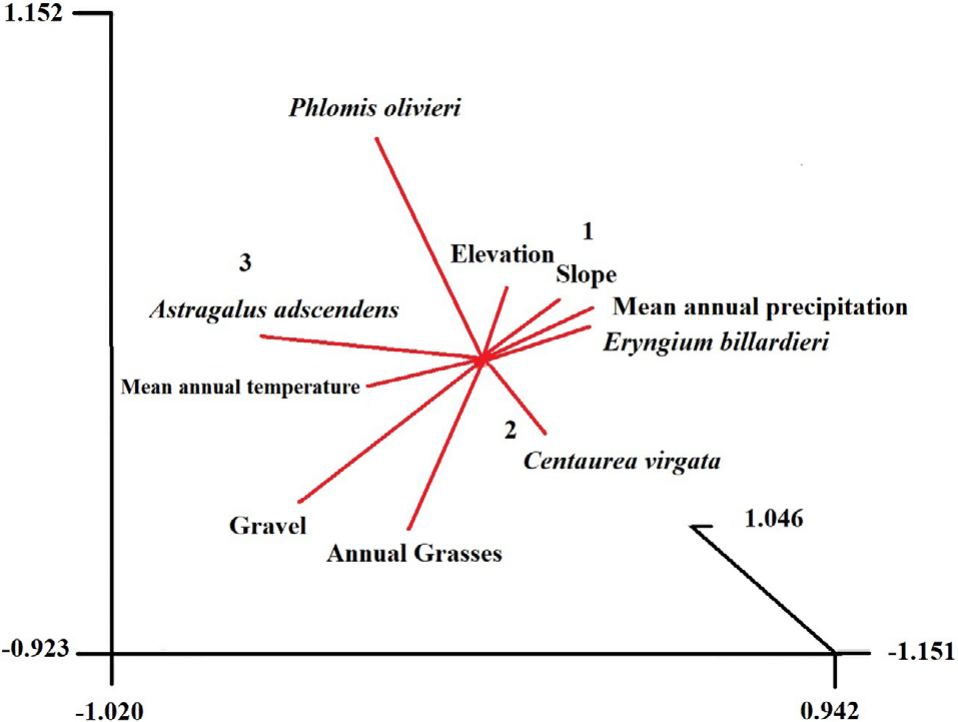
Principal axis correlation depicting the significant associations (*p* < 0.05) between five species vectors and five environmental variables. The correlations are visualized in a three‐dimensional nonparametric multidimensional scaling ordination, with centroids marking three distinct groups (SSH MDS, Bray and Courtis Metric, Cut‐off = 0.9, stress = 0.1408), using the FLEXIBLE UPGMA technique. Symbols denote groups generated by PATN, with each symbol representing sites sharing similar compositions.

As depicted in Figure 6, the analysis revealed significant correlations (*p* < 0.05) between 5 plant species and 7 environmental variables within the three‐dimensional NMDS ordination space. The results revealed three distinct groups. According to the PCC analysis results (Figure 6), Group One, characterized by abundant *Eryngium billardieri* and associated with higher mean annual precipitation, elevation, slope, and elevated nitrogen, and calcium content in surface soils. Group Two, dominated by *Centaurea virgata* , and Group Three, featuring *Astragalus adscendens*, are primarily associated with higher mean annual temperature and gravel content in the soil.

### 3.5 Analysis and Ranking of Invasive Species

The analysis conducted using the AHP method to identify the most hazardous invasive species revealed that the final weights assigned to the impact, invasion, and spread potential criteria were 0.66, 0.24, and 0.10, respectively (Table 5). These weights underscore the significance of these criteria in effectively evaluating the invasive species under scrutiny. The findings further elucidate that, based on the evaluated criteria, the ranking of invasive species risk positioned *Cousinia bachtiarica*, *Eryngium billardieri*, *Phlomis persica*, *Euphorbia decipiens*, and *Poa bulbosa* with weights of 0.36, 0.26, 0.14, 0.13, and 0.12, respectively. Notably, among the primary criteria, the impact criterion exerted the most substantial influence, while the spread potential criterion had the least impact on the assessment of invasive plant risk.

**TABLE 5:** Analytic hierarchy process analysis of invasive species risk factors.

Criteria	Weight	Subcriteria	Subcriteria weight	Options	Option Weight
Impact of invasive plants	0.66	Interference or blockage from entry of desired species	0.15	*Eryngium billardieri*	0.26
		Ecosystem damage	0.75	*Cousinia bachtiarica*	0.36
		Resistance to control	0.91		
Invasion	0.24	Adaptability	0.14	*Phlomis persica*	0.14
		Competitive strength	0.47		
		Reproduction and dispersion	0.37		
Spread potential	0.10	Occupied suitable habitats by invasive plants	0.75	*Euphorbia decipiens*	0.13
		Existence of these species in multiple countries	0.25	*Poa bulbosa*	0.12

## 4 Discussion

In this study, we investigated the relationship between the studied invasive species and environmental factors, identifying the influential factors in their presence. This involved examining the root system of the species and employing parametric and nonparametric multivariate analyses. Additionally, we prioritized invasive plant species under investigation by determining the indicators and criteria in collaboration with experts on invasive species.

### 4.1 Ecological Understanding of Invasive Species

Several soil characteristics, including nitrogen and calcium content in the soil surface, alongside physiographic factors such as elevation and slope, in conjunction with precipitation, emerged as the most significant contributors to the distribution of invasive species, as determined by both parametric and nonparametric ordination techniques. Consistent with in't Zandt et al. (2021), who reported a correlation between species abundance and soil nutrient levels across diverse ecosystems, our results confirm that soil properties are critical in shaping invasive species distribution. This highlights the consistency of our findings with prior research emphasizing soil attributes in determining plant distributions.

In addition to abiotic factors, biological and ecological processes may limit the establishment and survival of invasive species. For instance, *Poa bulbosa* , while drought‐tolerant, may face recruitment and germination challenges due to soil moisture and temperature fluctuations (Ofir and Kigel 2003). *Eryngium billardieri* and *Poa bulbosa* also encounter pollination and seed dispersal constraints, especially in environments lacking pollinator populations. Ehsani et al. (2013) similarly underscored the influence of environmental factors, such as soil acidity and nitrogen content, on *Poa bulbosa* 's establishment. Soil seed bank dynamics and potential pest pressures could further limit growth, particularly under degraded conditions.

Under high grazing pressure, less palatable invasive species may gain a competitive advantage over more palatable, productive, and perennial (3P) species. Livestock prefer palatable species, creating opportunities for invasive species like *Phlomis persica*, *Euphorbia decipiens*, and *Eryngium billardieri*, which are avoided due to their unpalatable, poisonous, or spiny nature, to proliferate. Rathfon et al. (2021) similarly noted that invasive species often exploit the weakened state of palatable species in heavily grazed areas. Future studies should examine biotic interactions, such as herbivory, competition, and disease susceptibility, to understand factors affecting invasive species more comprehensively.

While invasive species are commonly linked to negative impacts, some also have beneficial aspects. For example, *Eryngium billardieri* is harvested by local communities during flowering, dried, and mixed with forage to feed livestock in winter (Khademi et al. 2021). It also has medicinal properties, with studies highlighting its phenolic, flavonoid content, and antimicrobial activities (Daneshzadeh et al. 2020). Similarly, *Phlomis persica* exhibits antinociceptive effects (Sarkhail et al. 2003). These findings, supported by Marshall et al. (2012) and Martin et al. (2015), highlight the context‐dependent roles of certain invasive species, suggesting tailored management strategies over generalized approaches.

The novelty of this study lies in its integrated approach, combining species‐specific ecological traits with advanced ordination techniques. This dual‐method approach provides a nuanced understanding of environmental variables interacting with species traits, offering comprehensive insights into invasive species presence in semi‐arid rangelands. The application of both parametric and nonparametric methods enhances the rigor of findings, yielding robust insights into species‐environment relationships.

The PCA analysis revealed the ecological preferences of invasive species across three distinct habitats. *Cousinia bakhtiarica*, for instance, thrives in shale formations with relatively high sodium levels, suggesting some degree of salt tolerance. *Euphorbia decipiens*, according to Pahlavani (2007), thrives in limestone soils between 1900 and 3200 m, and the PCA results suggest similar environmental preferences for *Euphorbia decipiens* and *Phlomis persica*. *Poa bulbosa* and *Eryngium billardieri* correlated strongly with precipitation, elevation, slope, and soil calcium and magnesium levels, consistent with Pakzad et al. (2013), who found *Eryngium billardieri* thriving in cold climates with high precipitation, as observed in our study.

The findings suggest that the presence of invasive species in the surveyed rangelands reflects pre‐existing soil conditions rather than degradation induced by these species. While soil chemical properties like pH, potassium, and phosphorus were consistent across sites, variations in soil texture, electrical conductivity, and sodium concentration were noted, particularly in Noghan Olya rangeland. Jafari et al. (2004) similarly observed that soil texture and electrical conductivity can vary widely across rangelands, impacting species distribution. The adaptable root systems of invasive species, such as *Eryngium billardieri* and *Cousinia bachtiarica*, enable them to access nutrients effectively, allowing them to establish in degraded or poorly managed rangelands.

Predictive modeling techniques, like Bayesian Belief Networks (BBN), can offer valuable insights into invasive species distributions, especially in data‐limited settings. Martin et al. (2015) demonstrated BBN's utility for forecasting invasive species spread, integrating expert knowledge with empirical data. Applying similar modeling approaches in our study could enhance our understanding of invasive species distribution across semi‐arid regions.

The degradation of rangelands and the prevalence of *Euphorbia decipiens* and *Phlomis persica* may be due to habitat proximity to roads and villages, leading to severe grazing and trampling. Field observations confirmed livestock activities in these habitats, reducing the availability of palatable forage as unpalatable species proliferate.

Clustering and PCA results indicate that species within each group share similar tolerance ranges and thrive under comparable soil conditions. All plots at the Fereydunshahr site clustered together, with *Eryngium billardieri* and *Poa bulbosa* showing the highest canopy cover percentages. The NMDS analysis further revealed significant correlations (*p* < 0.05) between *Eryngium billardieri* canopy cover and variables like calcium content and elevation, affirming PCA and Kruskal–Wallis findings. This agreement among multivariate analyses strengthens the reliability of our results (Rencher 2002).

Soil emerges as a primary determinant of vegetation cover and density, reflecting cumulative environmental impacts over time. Changes in soil attributes can profoundly impact ecosystem functionality, with vegetation cover and soil composition intricately linked. This insight is particularly relevant for arid and semi‐arid rangelands, where disruptive dynamics shape ecosystems, underscoring the importance of effective management and conservation strategies.

### 4.2 Prioritization of Invasive Species

In the findings derived from prioritizing invasive plant species, the criteria concerning impacts, invasiveness, and potential for spread emerged as the most effective for evaluating the studied species. *Cousinia bachtiarica* was acknowledged as the most hazardous invasive species, whereas *Poa bulbosa* was deemed the least risky among the species under study. While invasiveness and potential for spread are important factors in assessing the threat posed by invasive species, impacts hold greater significance due to their direct implications for ecosystems, biodiversity, and human activities (Pimentel et al. 2005). The severity of impacts determines the urgency and prioritization of management efforts, making it a primary consideration in invasive species management strategies (Vilà et al. 2010; Perrings et al. 2005). Therefore, in our study, criteria related to impacts were given precedence in the prioritization of invasive plant species.

To ensure robust AHP analysis, we used Expert Choice software, which calculates criteria weights and assesses consistency ratios for logical coherence in pairwise comparisons. A consistency ratio under 0.1, as recommended by Saaty (1980), indicates reliable judgments. The criteria and weights for comparisons were based on expert opinions, reinforcing reliability. Additionally, we validated the model's rankings through field observations (Pheloung et al. 1999), where highly ranked species like *Cousinia bachtiarica* correlated with greater ecosystem impacts and resistance to control.

*Cousinia bachtiarica* is safeguarded against grazing due to its thorny and woody aerial parts, rendering it unpalatable for livestock. Its sharp thorns act as a deterrent, preventing grazing at any stage of its growth. Moreover, this plant disperses thorny seeds easily through the wind. In contrast, *Eryngium billardieri*, though initially spared from grazing during its early growth stages when its thorns are undeveloped, later encounters reduced forage availability due to its thorny nature. It boasts a robust root system and serves as a nectar source for bees. *Euphorbia decipiens*, characterized by its well‐established root system and nontoxic latex sap, faces minimal grazing pressure from sheep, the primary livestock in these regions, resulting in lower distribution and density compared to other studied plants. *Phlomis persica*, owing to its aromatic essence, is less appealing to livestock for grazing but possesses a sturdy root system and is renowned for its medicinal properties. Additionally, *Poa bulbosa* is grazed by livestock, although its short stature makes it less susceptible to grazing. Based on the findings, it is evident that as rangeland density increases, *Poa bulbosa* covers the soil surface like a sponge with its specific root system. During precipitation, these roots absorb moisture, impeding water infiltration into deeper soil layers. Consequently, plants with deeper roots struggle to absorb moisture, leading to their displacement from the rangeland surface and exacerbating soil erosion (Pierret et al. 2016).

### 4.3 Management Implications

The comprehensive ecological analysis conducted in this study provides valuable insights into the relationship between invasive plant species and their environment, essential for developing effective management strategies tailored to specific habitats and species. By prioritizing invasive plant species based on their impacts, invasiveness, and potential for spread, targeted control efforts can be implemented to maximize effectiveness and mitigate ecological and economic impacts. Tailored management approaches, such as preemptive removal, targeted grazing, and the promotion of competitive native species, can effectively control invasive plant populations while minimizing negative impacts on native ecosystems. Collaboration between stakeholders, including researchers, land managers, policymakers, and local communities, is crucial for effective invasive species management, fostering knowledge sharing, and leveraging collective expertise and resources.

Based on the results of the hierarchical analysis, *Cousinia bachtiarica* emerges as the most perilous invasive species among those studied, warranting heightened control and management measures in the study area. Given the plant's deterrent thorns, which discourage sheep grazing, employing goats for grazing during the nonwoody early season stages could prove beneficial. Alternatively, preemptive removal of the plant from rangelands before seeding could effectively impede its spread. Moreover, harnessing plants with robust competitive abilities could play a pivotal role in ecosystem modification, promoting the proliferation of desired species. These approaches are applicable to other species under scrutiny. Implementing integrated methods, such as initial mechanical treatments followed by controlled burning and subsequent targeted grazing by animals, may yield more favorable outcomes in curbing the spread of this invasive plant. Additionally, nurturing superior species with strong competitive traits, like perennial grasses and palatable and productive shrubs, alongside strategic grazing practices, enables tailored management. By adjusting grazing patterns based on plant composition, animal species, herd selection, population dynamics, and seasonal variations, undue pressure on undesirable rangeland species can be alleviated, while favorably grazing superior species.

Following the ecological assessments of the habitats under investigation, it can be concluded that rangelands predominantly occupied by *Phlomis persica* and *Euphorbia decipiens* species provide an environmentally favorable environment for direct seeding projects. In contrast, areas with a significant presence of *Eryngium billardieri* are less suitable for seeding, highlighting the importance of careful attention to local soil and rangeland conditions.

Through the ecological analysis of invasive species, valuable information about influential environmental factors and their associated species can be obtained, which can be utilized for prioritization and control purposes. While trait‐based studies have provided valuable insights into *Poa bulbosa* (Ofir and Kigel 2003, 2006), similar studies for other invasive species, such as *Eryngium billardieri*, *Euphorbia decipiens*, *Phlomis persica*, and *Cousinia bachtiarica*, are rare and focus primarily on medicinal uses or taxonomic classification rather than ecological traits. This gap highlights the need for further research to better understand the ecological roles of these species, which can help inform more precise control strategies. Analyzing ecological data of invasive species, along with their management information, can improve the interpretation regarding the distribution, prioritization, and control of these species (Gurevitch and Padilla 2004).

## 5 Conclusions

This study has significantly advanced the understanding of ecological dynamics and management strategies for invasive plant species in the semi‐arid rangelands of Iran. One of the primary achievements of this research is the development of a robust framework for analyzing the relationship between invasive species and environmental factors such as soil characteristics, elevation, slope, and precipitation, which has led to a more nuanced understanding of how these species establish and spread in sensitive ecosystems.

By employing the AHP approach to prioritize invasive species, the study has enabled more targeted management efforts, ensuring that control measures are focused on species with the greatest ecological and economic impacts. This prioritization allows land managers to allocate resources effectively, addressing the most pressing invasion threats first.

Additionally, the study highlights the importance of tailoring management strategies to habitat‐specific conditions. The findings emphasize that the ecological preferences of invasive species vary based on environmental factors, reinforcing the need for context‐specific management approaches. This ensures that interventions are more effective and sustainable over time.

Overall, this research provides a foundation for adaptive management strategies that not only mitigate the immediate effects of invasive species but also address their long‐term potential for spread. Continued research and collaboration will be essential for preserving the native biodiversity and ecosystem functions of these rangelands, ensuring that Iran's semi‐arid ecosystems continue to provide essential ecosystem services and support human well‐being.

## Author Contributions

**Hossein Bashari:** conceptualization (equal), data curation (equal), formal analysis (equal), investigation (equal), methodology (equal), project administration (equal), resources (equal), software (equal), supervision (equal), validation (equal), visualization (equal), writing – original draft (equal), writing – review and editing (lead). **Fereshteh Bazgir:** data curation (equal), formal analysis (equal), investigation (equal), methodology (equal), project administration (lead), resources (equal), validation (equal), visualization (equal), writing – original draft (equal), writing – review and editing (equal). **Mohammad Reza Vahabi:** conceptualization (equal), formal analysis (equal), investigation (equal), methodology (equal), supervision (equal), writing – review and editing (equal).

## Conflicts of Interest

The authors declare no conflicts of interest.

## Supporting information


Data S1.

## Data Availability

The soil and vegetation data supporting this study have been provided as supporting material with the manuscript submission. Additional details can be made available upon reasonable request.

## References

[ece371287-bib-0001] Bahrami, M. , F. Sarmadian , and E. Pazira . 2024. “Integrating AHP (Analytic Hierarchy Process) and GIS (Geographic Information System) for Precision Land Use Planning and Ecological Capacity Assessment in Alborz Province, Iran.” EQA‐International Journal of Environmental Quality 64: 48–67.

[ece371287-bib-0003] Belbin, L. 2003. PATN. A Software for Extracting and Displaying Patterns in Any Type of Complex (Multivariate) Data. Griffith University and the University of Queensland, Brisbane.

[ece371287-bib-0004] Bray, J. R. , and J. T. Curtis . 1957. “An Ordination of the Upland Forest Communities of Southern Wisconsin.” Ecological Monographs 27, no. 4: 325–349.

[ece371287-bib-0005] Carter, M. R. , and E. G. Gregorich . 2008. Soil Sampling Methods of Analysis. 2nd ed, 1224. CRC Press.

[ece371287-bib-0006] Cohen, J. G. , H. D. Enander , T. J. Bassett , C. M. Wilton , and A. A. Cole‐Wick . 2024. “Using GIS‐Based Multicriteria Decision Analysis to Prioritize Invasive Plant Treatment: A Creative Solution for a Pernicious Problem.” Ecological Modelling 495: 110807.

[ece371287-bib-0007] Convention on Biological Diversity (CBD) . 2014. Global Biodiversity Outlook 4. Secretariat of the Convention on Biological Diversity, Montréal.

[ece371287-bib-0008] Daneshzadeh, M. S. , H. Abbaspour , L. Amjad , and A. M. Nafchi . 2020. “An Investigation on Phytochemical, Antioxidant and Antibacterial Properties of Extract From *Eryngium Billardieri* F. Delaroche.” Journal of Food Measurement and Characterization 14: 708–715.

[ece371287-bib-0009] Dukes, J. S. , and H. A. Mooney . 2004. “Disruption of Ecosystem Processes in Western North America by Invasive Species.” Revista Chilena de Historia Natural 77, no. 3: 411–437. 10.4067/S0716-078X2004000300003.

[ece371287-bib-0010] Early, R. , B. A. Bradley , J. S. Dukes , et al. 2016. “Global Threats From Invasive Alien Species in the Twenty‐First Century and National Response Capacities.” Nature Communications 7, no. 1: 12485. 10.1038/ncomms12485. PMC4996970 27549569

[ece371287-bib-0011] Ehsani, A. , H. Yeganeh , A. Sour , K. F. Saghafi , G. A. Abarsaji , and H. Akbarpour . 2013. “The Study of Phenology Stage of *Poa Bulbosa* in Sei‐Steppe Regions (Golestan, Khorasan Razavi).” Journal of Plant Environmental Physiology 8, no. 1: 17–27.

[ece371287-bib-0012] Elith, J. 2017. “Predicting Distributions of Invasive Species.” In Invasive Species: Risk Assessment and Management, edited by A. P. Robinson , T. Walshe , M. A. Burgman , and M. Nunn , 93–129. Cambridge University Press.

[ece371287-bib-0013] Ewel, J. J. , and F. E. Putz . 2004. “A Place for Alien Species in Ecosystem Restoration.” Frontiers in Ecology and the Environment 2, no. 7: 354–360.

[ece371287-bib-0014] Foley, J. A. , R. DeFries , G. P. Asner , et al. 2005. “Global Consequences of Land Use.” Science 309, no. 5734: 570–574. 10.1126/science.1111772. 16040698

[ece371287-bib-0015] Froment, A. 1972. “Soil Respiration in a Mixed Oak Forest.” Oikos 23, no. 2: 273–277. 10.2307/3543417.

[ece371287-bib-0017] Gioria, M. , V. Jarošík , and P. Pyšek . 2014. “Impact of Invasions by Alien Plants on Soil Seed Bank Communities: Emerging Patterns.” Perspectives in Plant Ecology, Evolution and Systematics 16, no. 3: 132–142.

[ece371287-bib-0018] Grošelj, P. , M. Zandebasiri , and Š. Pezdevšek Malovrh . 2023. “Evaluation of the European Experts on the Application of the AHP Method in Sustainable Forest Management.” Environment, Development and Sustainability 26: 1–27.

[ece371287-bib-0019] Gurevitch, J. , and D. K. Padilla . 2004. “Are Invasive Species a Major Cause of Extinctions?” Trends in Ecology & Evolution 19, no. 9: 470–474. 16701309 10.1016/j.tree.2004.07.005

[ece371287-bib-0020] in't Zandt, D. , T. Herben , A. van den Brink , E. J. Visser , and H. de Kroon . 2021. “Species Abundance Fluctuations Over 31 Years Are Associated With Plant–Soil Feedback in a Species‐Rich Mountain Meadow.” Journal of Ecology 109, no. 3: 1511–1523.

[ece371287-bib-0021] Jafari, M. , M. Z. Chahouki , A. Tavili , H. Azarnivand , and G. Z. Amiri . 2004. “Effective Environmental Factors in the Distribution of Vegetation Types in Poshtkouh Rangelands of Yazd Province (Iran).” Journal of Arid Environments 56, no. 4: 627–641. 10.1016/S0140-1963(03)00077-6.

[ece371287-bib-0022] Khademi, T. , M. Rostampour , and M. Saghari . 2021. “Nutritive Value of Dominant Rangeland Plant Species in Kaja and Chahno, Ferdows, South Khorasan.” Rangelands 15, no. 4: 649–664.

[ece371287-bib-0023] Khajeddin, S. J. , and H. Yeganeh . 2008. “Plant Communities of the Karkas Hunting‐Prohibited Region, Isfahan‐Iran.” Plant, Soil and Environment 54, no. 8: 347–358.

[ece371287-bib-0024] Khedrigharibvand, H. , H. Azadi , H. Bahrami , et al. 2018. “Sustainable Rangeland Management in Southwest Iran: Application of the AHP‐TOPSIS Approach in Ranking Livelihood Alternatives.” Rangeland Journal 40, no. 6: 603–614. 10.1071/RJ17038.

[ece371287-bib-0025] Kruskal, W. H. , and W. A. Wallis . 1952. “Use of Ranks in One‐Criterion Variance Analysis.” Journal of the American Statistical Association 47, no. 260: 583–621. 10.1080/01621459.1952.10483441.

[ece371287-bib-0026] Mack, R. N. , D. Simberloff , W. Mark Lonsdale , H. Evans , M. Clout , and F. A. Bazzaz . 2000. “Biotic Invasions: Causes, Epidemiology, Global Consequences, and Control.” Ecological Applications 10, no. 3: 689–710.

[ece371287-bib-0027] Marshall, V. M. , M. M. Lewis , and B. Ostendorf . 2012. “Buffel Grass ( *Cenchrus ciliaris* ) as an Invader and Threat to Biodiversity in Arid Environments: A Review.” Journal of Arid Environments 78: 1–12.

[ece371287-bib-0028] Martin, T. G. , H. Murphy , A. Liedloff , et al. 2015. “Buffel Grass and Climate Change: A Framework for Projecting Invasive Species Distributions When Data Are Scarce.” Biological Invasions 17: 3197–3210.

[ece371287-bib-0029] Master, J. , I. Qayim , D. Setiadi , and N. Santoso . 2020. “Autecology of *Melastoma Malabathricum*, an Invasive Species in the Way Kambas National Park, Indonesia.” Biodiversitas Journal of Biological Diversity 21, no. 5: 2303–2310.

[ece371287-bib-0030] Mirdavoodi, H. , M. R. Marvi Mohadjer , G. Zahedi Amiri , and V. Etemad . 2013. “Disturbance Effects on Plant Diversity and Invasive Species in Western Oak Communities of Iran (Case Study: Dalab Forest, Ilam).” Iranian Journal of Forest and Poplar Research 21, no. 1: 1–15.

[ece371287-bib-0031] Mostert, E. , M. Gaertner , P. M. Holmes , P. J. O'Farrell , and D. M. Richardson . 2018. “A Multi‐Criterion Approach for Prioritizing Areas in Urban Ecosystems for Active Restoration Following Invasive Plant Control.” Environmental Management 62, no. 6: 1150–1167. 10.1007/s00267-018-1103-9. 30242527

[ece371287-bib-0032] Mukundi, J. B. , S. W. Waweru , and P. K. Ndang'ang'a . 2024. “Implication of Abandoned Post‐Quarry Sites on Avifauna Composition for Strategic Landscape Restoration in Ndarugu, Kiambu, Kenya.” Land Degradation & Development 35, no. 6: 2022–2032.

[ece371287-bib-0033] Nielsen, A. M. , and S. Fei . 2015. “Assessing the Flexibility of the Analytic Hierarchy Process for Prioritization of Invasive Plant Management.” NeoBiota 27: 25–36. 10.3897/neobiota.27.4919.

[ece371287-bib-0034] Novak, S. J. , and A. Y. Welfley . 1997. “Genetic Diversity in the Introduced Clonal Grass *Poa Bulbosa* (Bulbous Bluegrass).” Northwest Science 71, no. 4: 271–280.

[ece371287-bib-0035] Ofir, M. , and J. Kigel . 2003. “Variation in Onset of Summer Dormancy and Flowering Capacity Along an Aridity Gradient in *Poa bulbosa* L., a Geophytic Perennial Grass.” Annals of Botany 91, no. 3: 391–400. 12547692 10.1093/aob/mcg026 PMC4244964

[ece371287-bib-0036] Ofir, M. , and J. Kigel . 2006. “Opposite Effects of Daylength and Temperature on Flowering and Summer Dormancy of *Poa bulbosa* .” Annals of Botany 97, no. 4: 659–666. 16467351 10.1093/aob/mcl021 PMC2803668

[ece371287-bib-0037] Oh, M. , Y. Heo , E. J. Lee , and H. Lee . 2021. “Major Environmental Factors and Traits of Invasive Alien Plants Determining Their Spatial Distribution.” Journal of Ecology and Environment 45: 1–10.

[ece371287-bib-0038] Pahlavani, A. 2007. “Notes on Some Species of the Genus Euphorbia in Iran.” Rostaniha 8, no. 2: 89–103.

[ece371287-bib-0039] Pakzad, Z. , M. Raeini Sarjaz , and M. Khodagholi . 2013. “Evaluation of the Effects of Climate Factors on Distribution of the Habitats of Astragalus Adscendens in Isfahan Province.” Iranian Journal of Range and Desert Research 20, no. 1: 199–212.

[ece371287-bib-0040] Perrings, C. , K. Dehnen‐Schmutz , J. Touza , and M. Williamson . 2005. “How to Manage Biological Invasions Under Globalization.” Trends in Ecology & Evolution 20, no. 5: 212–215. 16701371 10.1016/j.tree.2005.02.011

[ece371287-bib-0041] Peters, K. , L. Breitsameter , and B. Gerowitt . 2014. “Impact of Climate Change on Weeds in Agriculture: A Review.” Agronomy for Sustainable Development 34, no. 4: 707–721. 10.1007/s13593-014-0245-2.

[ece371287-bib-0042] Pheloung, P. C. , P. A. Williams , and S. R. Halloy . 1999. “A Weed Risk Assessment Model for Use as a Biosecurity Tool Evaluating Plant Introductions.” Journal of Environmental Management 57, no. 4: 239–251.

[ece371287-bib-0043] Pierret, A. , J. L. Maeght , C. Clément , J. P. Montoroi , C. Hartmann , and S. Gonkhamdee . 2016. “Understanding Deep Roots and Their Functions in Ecosystems: An Advocacy for More Unconventional Research.” Annals of Botany 118, no. 4: 621–635. 27390351 10.1093/aob/mcw130 PMC5055635

[ece371287-bib-0044] Pimentel, D. , R. Zuniga , and D. Morrison . 2005. “Update on the Environmental and Economic Costs Associated With Alien‐Invasive Species in the United States.” Ecological Economics 52, no. 3: 273–288.

[ece371287-bib-0045] Pyšek, P. , P. E. Hulme , D. Simberloff , et al. 2020. “Scientists' Warning on Invasive Alien Species.” Biological Reviews 95, no. 6: 1511–1534. 10.1111/brv.12627. 32588508 PMC7687187

[ece371287-bib-0046] Rathfon, R. A. , S. M. Greenler , and M. A. Jenkins . 2021. “Effects of Prescribed Grazing by Goats on Non‐Native Invasive Shrubs and Native Plant Species in a Mixed‐Hardwood Forest.” Restoration Ecology 29, no. 4: e13361. 10.1111/rec.13361.

[ece371287-bib-0047] Rencher, A. C. 2002. Methods of Multivariate Analysis, 727. John Wiley & Sons.

[ece371287-bib-0048] Saaty, T. L. 1980. The Analytic Hierarchy Process. Mcgraw Hill.

[ece371287-bib-0049] Sala, O. E. , F. I. I. I. Stuart Chapin , J. J. Armesto , et al. 2000. “Global Biodiversity Scenarios for the Year 2100.” Science 287, no. 5459: 1770–1774. 10710299 10.1126/science.287.5459.1770

[ece371287-bib-0050] Sardinha, M. , T. Müller , H. Schmeisky , and R. G. Joergensen . 2003. “Microbial Performance in Soils Along a Salinity Gradient Under Acidic Conditions.” Applied Soil Ecology 23, no. 3: 237–244. 10.1016/S0929-1393(03)00027-1.

[ece371287-bib-0200] Sarkhail, P. , M. Abdollahi , and A. Shafiee . 2003. “Antinociceptive Effect of Phlomis olivieri Benth., Phlomis anisodonta Boiss. and Phlomis persica Boiss. Total Extracts.” Pharmacological Research 48, no. 3: 263–266. 12860444 10.1016/s1043-6618(03)00151-8

[ece371287-bib-0100] Sefidkon, F. , M. Dabiri , and A. Alamshahi . 2004. “Chemical Composition of the Essential Oil of Eryngium billardieri F. Delaroche from Iran.” Journal of Essential Oil Research 16, no. 1: 42–43.

[ece371287-bib-0300] Shakeri Broojeni, N. , H. Bashari , and M. Tarkesh . 2014. “Identifying Grazing Indicator Species Using Gradient Analysis Approach in Semi‐Steppe Range Lands of Feridan‐Isfahan.” Journal of Rangeland 8, no. 2: 201–212. (In Farsi).

[ece371287-bib-0052] Simberloff, D. , L. Souza , M. A. Nunez , M. N. Barrios‐Garcia , and W. A. Bunn . 2012. “The Natives Are Restless, but Not Often and Mostly When Disturbed.” Ecology 93, no. 3: 598–607. 10.1890/11-1232.1. 22624214

[ece371287-bib-0053] Strayer, D. L. 2012. “Eight Questions About Invasions and Ecosystem Functioning.” Ecology Letters 15, no. 10: 1199–1210. 22694728 10.1111/j.1461-0248.2012.01817.x

[ece371287-bib-0054] ter Braak, C. J. F. , and P. Šmilauer . 2002. CANOCO Reference Manual and CanoDraw for Windows User's Guide: Software for Canonical Community Ordination (Version 4.5). Microcomputer Power.

[ece371287-bib-0055] Thammanu, S. , D. Marod , H. Han , et al. 2021. “The Influence of Environmental Factors on Species Composition and Distribution in a Community Forest in Northern Thailand.” Journal of Forestry Research 32: 649–662.

[ece371287-bib-0056] Vilà, M. , C. Basnou , P. Pyšek , et al. 2010. “How Well Do We Understand the Impacts of Alien Species on Ecosystem Services? A Pan‐European, Cross‐Taxa Assessment.” Frontiers in Ecology and the Environment 8, no. 3: 135–144. 10.1890/080083.

[ece371287-bib-0057] Vilà, M. , J. L. Espinar , M. Hejda , et al. 2011. “Ecological Impacts of Invasive Alien Plants: A Meta‐Analysis of Their Effects on Species, Communities and Ecosystems.” Ecology Letters 14, no. 7: 702–708. 21592274 10.1111/j.1461-0248.2011.01628.x

[ece371287-bib-0058] Weaver, R. W. , J. S. Angel , and P. S. Bottomley . 1994. Methods of Soil Analysis: Microbiological and Biochemical Properties, 1152. Soil Society of America.

