# Correction to “Prioritizing the Risk of Multiple Invasive Species in the Semiarid Rangelands of Iran: An Ecological Approach to Multicriteria Decision‐Making”

**DOI:** 10.1002/ece3.71601

**Published:** 2025-07-22

Bashari, H., Bazgir, F. and Vahabi, M.R. (2025), Prioritizing the Risk of Multiple Invasive Species in the Semiarid Rangelands of Iran: An Ecological Approach to Multicriteria Decision‐Making. *Ecol Evol*, 15: e71287. https://doi.org/10.1002/ece3.71287

In Figure 3 of the published article, an earlier version of the figure was mistakenly included. This version contains several taxonomic inaccuracies:

- **Incorrect author citations**:
  ○ *Eryngium billardieri* Boiss. → should be *Eryngium billardieri* F. Delaroche
  ○ *Cousinia bachtiarica* Boiss. → should be *Cousinia bachtiarica* Boiss. & Hausskn.
  ○ *Poa bulbosa* Boiss. → should be *Poa bulbosa* L.
  ○ *Euphorbia decipiens* Boiss. → should be *Euphorbia deceipiens* Boiss. & Bushe.
- **Duplicate entries**:
  ○ *Phlomis persica* and *Euphorbia deceipiens* appear twice.

**FIGURE 3:**
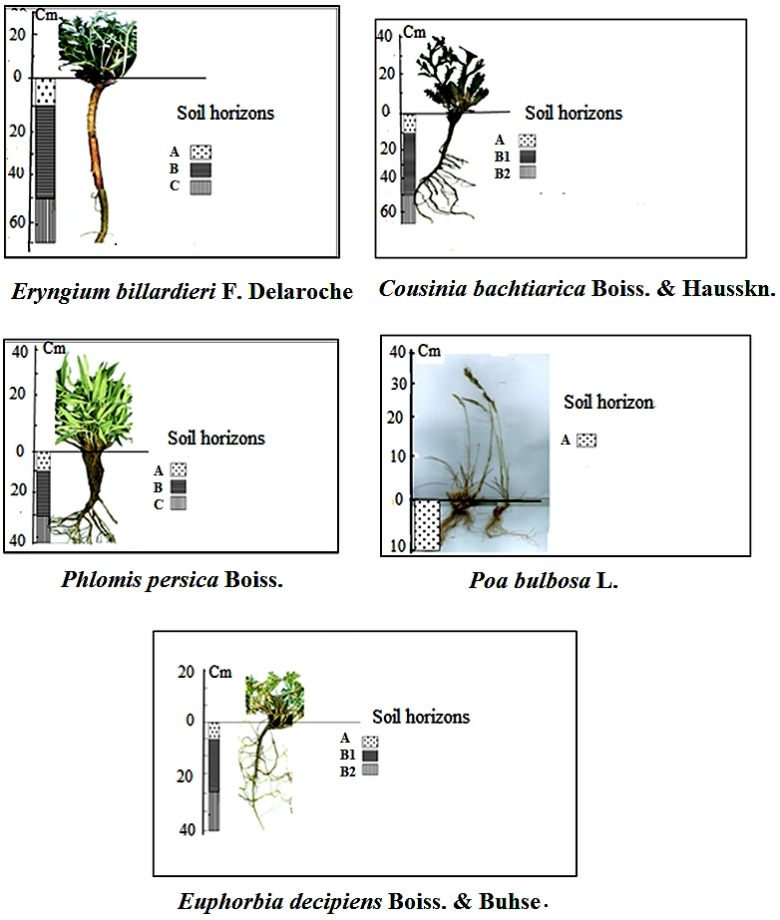
Above‐ground and below‐ground images of the invasive species under study, complemented by soil horizon details.

The correct version of Figure 3 is displayed below:

The online version of this article has been corrected accordingly.

We apologize for this error.

